# Supplementary material for: Return to 1616: Multispecies Fauna Reconstruction Requires Thinking Outside the Box
Source: Animals (Basel). 2023 Aug 30;13(17):2762. doi: 10.3390/ani13172762 (PMC10486414; doi:10.3390/ani13172762)
Supplement: Supplementary file 1 [file animals-13-02762-s001.zip › animals-2521898-supplementary.pdf]

Table S1. List of terrestrial mammal species confirmed or presumed to have previously occurred on Dirk Hartog Island in historical times, as well as two bird species known to have become locally extinct (Status refers to if a species has been confirmed to occur or have occurred on the island (CE, confirmed and extant; CN, confirmed and not planned for translocation; CP, confirmed and planned for translocation; CT, confirmed and translocated; UN, unconfirmed and not planned for translocation; UT, unconfirmed and translocated); Listing refers to conservation status under the Environmental Protection and Biodiversity Conservation (EPBC) Act 1999 (V, Vulnerable; E, Endangered, N, not listed); Guild refers to the dietary guild each species can be classified into (H, herbivore; I, invertivore; O, omnivore)).

Common Name	Scientific Name	Status	Listing	Guild	Comments
Brush-tailed mulgara	*Dasycercus blythi*	CT*	N	O	Subfossil evidence (Baynes 1990, 2008)
Chuditch	*Dasyurus geoffroii*	CP	V	O†	Subfossil evidence (Baynes 1990, 2008)
Dibbler	*Parantechinus apicalis*	CT	E	O	Subfossil evidence (Baynes 1990, 2008)
Little long-tailed dunnart	*Sminthopsis dolichura*	CE	N	I	Extant
Shark Bay bandicoot	*Perameles bougainville*	CT	E	O	Subfossil evidence (Baynes 1990, 2008)
Boodie	*Bettongia lesueur*	CP	V	O	Subfossil evidence (Baynes 1990, 2008)
Woylie	*Bettongia penicillata*	CP	E	O	Subfossil evidence (Baynes 1990, 2008)
Banded hare-wallaby	*Lagostrophus fasciatus*	UT	V	H	Anecdotal evidence (e.g. Orchard and Orchard 2018)
Rufous hare-wallaby	*Lagorchestes hirsutus*	UT	V	H	Anecdotal evidence (e.g. Shortridge 1909)
Rakali	*Hydromys chrysogaster*	UN	N	O	Circumstantial evidence (Morris et al. 2017)
Greater Stick-nest rat	*Leporillus conditor*	CT	V	H	Subfossil evidence (Baynes 1990, 2008)
Ash-grey mouse	*Pseudomys albocinereus*	CE	N	O	Extant
Desert mouse	*Pseudomys desertor*	CP	N	H	Subfossil evidence (Baynes 2008)
Shark Bay mouse	*Pseudomys gouldii*	CT	V	O	Subfossil evidence (Baynes 1990, 2008)
Sandy inland mouse	*Pseudomys hermannburgensis*	CE	N	O	Extant
Heath mouse	*Pseudomys shortridgei*	CP	E	O	Subfossil evidence (Baynes 1990, 2008)
Lesser long-eared bat	*Nyctophilus geoffroyi*	CE	N	I	Extant
Finlayson's cave bat	*Vespadelus finlaysoni*	CE	N	I	Extant
Rock parrot	*Neophema petrophila*	CN	N	H	Last recorded in 1916 (Carter 1917; Burbidge and George 1972)
Western grasswren	*Amytornis textilis*	CT	N	O	Specimens collected in 1916 (Carter 1917)

* *Dasycercus* sp. that occurred on Dirk Hartog Island presumed to be this taxon but has not been confirmed; † although omnivorous, this species is considered to be the terrestrial apex predator on Dirk Hartog Island.
